# Genetic diversity and population structure of *Saccharum* hybrids

**DOI:** 10.1371/journal.pone.0289504

**Published:** 2023-08-15

**Authors:** María Francisca Perera, Santiago Ostengo, Andrea Natalia Peña Malavera, Thiago Willian Almeida Balsalobre, Guilherme Dias Onorato, Aldo Sergio Noguera, Hermann Paulo Hoffmann, Monalisa Sampaio Carneiro

**Affiliations:** 1 Instituto de Tecnología Agroindustrial del Noroeste Argentino (ITANOA), Estación Experimental Agroindustrial Obispo Colombres (EEAOC)—Consejo Nacional de Investigaciones Científicas y Técnicas (CONICET), Las Talitas, Tucumán, Argentina; 2 Sugarcane Breeding Program of RIDESA/UFSCar, Araras, São Paulo, Brazil; 3 Departamento de Biotecnologia e Producão Vegetal e Animal, Centro de Ciências Agrárias, Universidade Federal de São Carlos (UFSCar), Araras, São Paulo, Brazil; ICAR - Indian Institute of Wheat and Barley Research, INDIA

## Abstract

Sugarcane breeding programs incorporate foreign material to broaden the genetic base, expanding the gene pool. In South America, the Inter-university Network for the Development of the Sugarcane Industry (RIDESA) and Estación Experimental Agroindustrial Obispo Colombres (EEAOC) sugarcane breeding programs from Brazil and Argentina, respectively, have never exchanged materials. In that sense, the knowledge of the genetic diversity and population structure among sugarcane genotypes of both germplasm banks, determined in a reliable way through their molecular profiles, will provide valuable information to select the best parental accessions for crossing aimed at the efficient introgression of desirable alleles. For that, the aim was to determine the genetic diversity and population structure of 96 *Saccharum* commercial hybrids from RIDESA and EEAOC sugarcane breeding programs by using TRAP, SSR and markers related to disease resistance (e.g. *Bru1* and G1). Genetic structure was determined through genetic similarity analysis, analysis of molecular variance (AMOVA), Multidimensional scaling (MDS), and a Bayesian method. Average PIC values were 0.25 and 0.26, Ho values were 0.24 and 0.28, and He values were 0.25 and 0.28, for TRAP and SSR primers, respectively. Genetic similarity, MDS, and analysis of structure revealed that Brazilian and Argentinean genotypes clustered in two groups clearly differentiated, whereas AMOVA suggested that there is more variability within programs than between them. Regarding *Bru1* markers, Brazilian genotypes showed high frequency of haplotype 1 (71.4%) whereas Argentinean genotypes showed high frequency of haplotype 4 (80.8%); haplotypes 1 and 4 are indicated for the presence and absence of the brown rust resistance gene (*Bru1*), respectively. Respecting the G1 marker, most of the evaluated genotypes (60.4%) showed the presence of the fragment, in a similar proportion for genotypes of both programs. In conclusion, the exchange of materials, at least the most diverse genotypes, between RIDESA and EEAOC breeding programs will allow extending the genetic base of their germplasm banks, and the knowledge of genetic diversity will help breeders to better manage crosses, increasing the probability of obtaining more productive varieties.

## Introduction

Sugarcane has a worldwide economic importance not only for food but also for ethanol production, electricity cogeneration, and the obtention of other sub-products. In sugarcane breeding, higher rates of biomass and/or sucrose production can be obtained through better management of genetic resources present in germplasm banks and core collections [1]. In order to broaden the genetic base, i.e. expanding the gene pool, breeding programs must incorporate foreign material. In South America, the Inter-university Network for the Development of the Sugarcane Industry (RIDESA) and Estación Experimental Agroindustrial Obispo Colombres (EEAOC) sugarcane breeding programs from Brazil and Argentina, respectively, have never exchanged materials. RIDESA is a partnership of 10 Brazilian Federal Universities with the purpose of developing improved sugarcane varieties and producing the varieties with the abbreviation RB. A public-private partnership was then established between RIDESA and companies from the national sugarcane sector, which started to provide research funding. In 30 years, RIDESA released 75 RB varieties. Several contributions were made by the network; in the Central-South region, for example, early-maturing varieties that came to stand out were released (RB835054, RB835486, RB855156, RB855453 and RB855536) [2]. Nowadays, RB sugarcane varieties are currently planted in 56% of the area cultivated with sugarcane in Brazil.

Among the three sugarcane breeding programs in Argentina, EEAOC produced TUC varieties. Such is its importance for Tucumán, the main sugar-producing province contributing more than 66% of the total production of the country, that in the last 12 years, it has released eight new varieties. Even more, the last varietal census revealed that more than 98% of the sugarcane area in Tucumán is planted with varieties released by this breeding program [3]. The EEAOC breeding program maintained an active variety exchange with sugarcane breeding programs of Florida (USDA-ARS, Sugarcane Field Station, Canal Point) and Louisiana (Louisiana State University Agricultural Center and USDA-ARS, Sugarcane Research Unit, Houma). Historically, materials from the USA breeding programs have shown good adaptation to Tucumán climatic and soil conditions, so they constitute the main source of foreign parents [4]. Thus, it is a challenge to exchange materials between UFSCar/RIDESA and EEAOC breeding programs. However, the previous knowledge of the genetic diversity and population structure among sugarcane genotypes of both germplasm banks will provide valuable information to select the best parental accessions for crossing aimed at efficient introgression of desirable alleles.

Usually, parents are selected based on phenotypic characteristics such as yield performance, sugar content, disease resistance, and progeny performance [4]. As phenotypic traits are influenced by environmental effects, the molecular profile could be used to identify in a more reliable way better combinations between accessions for crossings and to estimate genetic diversity. Different types of molecular markers have been used for this kind of studies in sugarcane germplasm collections (RFLP, AFLP, RAPD, SSR, ISSR, STMS, SNPs) [5]. However, in order to screen sugarcane genotypes in functional regions of their genomes, Target Region Amplified Polymorphism (TRAP) markers [6] are an excellent option. Specifically, the use of TRAP markers based on genes that governed metabolic pathways of sucrose and lignin synthesis could aid to find new allelic variants considering that increasing sugar content is one of the main goals of breeding programs [7], while decreasing lignin content may facilitate cellulose saccharification for energy cane purpose [8]. Besides, Simple Sequence Repeats from Expressed Sequence Tag (EST-SSR) molecular markers could be used to characterize genotypes into functional regions of the genome [1]. TRAP and EST-SSR and even other functional markers have been successfully used for diversity studies in sugarcane [5]. Whereas some studies with different marker technologies revealed that breeding programs should increase the genetic diversity of their collections to meet the demand of sugarcane cultivation for sugar and bioenergy use, others suggested that the observed genetic diversity of the ancestors has been captured in sugarcane germplasm [5]. Regarding the germplasm bank of RB varieties, SSR revealed an increase in genetic similarity in the 1970s due to interbreeding with few parents; however, after the 1990s, with the introduction of new parents, there was a decrease in genetic similarity levels [9]. For EEAOC, a previous study using AFLP to characterize only a few parents, revealed high genotype similarities [10]. Furthermore, the most effective method to control several sugarcane diseases is the use of resistant commercial varieties. In the case of brown rust, an important sugarcane disease in many production areas worldwide caused by *Puccinia melanocephala*, the resistance in modern sugarcane cultivars relies essentially on a single copy allele, called *Bru1* [11]. Two flanking molecular markers (R12H16 and 9O20-F4-RsaI) represent valuable diagnostic markers for the presence of *Bru1* since they allow predicting a resistant behaviour of any modern sugarcane cultivar. In a previous study where sugarcane accessions of EEAOC´s bank were evaluated under field infection, only 16.3% of resistant genotypes to brown rust harboured the *Bru1* gene [12], proposing the existence of other alternative resistant sources, recently confirmed by Chaves et al. [13]. Whereas a high frequency of *Bru1* (73.5%) among brazilian cultivars suggested this gene is the prevalent source of brown rust resistance in Brazilian sugarcane breeding programmes [14], reinforcing the importance of germplasm exchange between both programs.

Regarding orange rust, another major disease impacting sugarcane production worldwide caused by *P*. *kuehnii*, a PCR-based resistance gene-derived maker, G1 was developed and it can be effectively utilized in sugarcane breeding programs to facilitate the selection process [15]. For example, the presence of this marker was tested in some Brazilian cultivars, it revealed 71.43% of efficiency in predicting the resistant phenotype considering cultivars with mean severity up to 3, in a scale that ranged from 1 (plants without symptoms) to 9 (highly susceptible plants) [16]. In Argentinean germplasm the presence of this marker has not yet been determined due to until now orange rust is not present in the main sugarcane producing area.

Considering the aforementioned results with molecular marker to estimate diversity and the detection of markers associated to disease resistance in both breeding programs, the aim of the present work was to determine the genetic diversity and population structure of 96 *Saccharum* commercial hybrids mainly from UFSCar/RIDESA and EEAOC sugarcane breeding programs in Brazil and Argentina, respectively, by using TRAP markers anchored in sucrose and lignin metabolism and drought tolerance genes, SSR and markers associated to disease resistance. Considering as it was aforementioned that these two breeding programs have never exchanged their materials, they probably do not share several alleles of their gene pools. For that, the exchange of genotypes would extend the genetic base of their respective germplasm banks and allow the introgression of new desirable alleles considering that the results are based on functional molecular markers and markers associated with disease resistance. This is the first time that this kind of comparative study is performed between both sugarcane breeding programs, reinforcing the advantage of exchanging materials, at least the most diverse genotypes.

## Materials and methods

### Plant material and DNA extraction

A total of 96 *Saccharum* spp. commercial hybrids were used in this study (S1 Table). Forty-nine genotypes belong to RIDESA active germplasm bank are being maintained at the Agricultural Science Center of the Federal University of São Carlos (UFSCar) in Araras City, São Paulo State, Brazil. The other 47 genotypes belong to the EEAOC germplasm collection that mainly involves advanced clones and commercial varieties of national and foreign origin that it is maintained in the experimental field of the EEAOC Central Research Station located in Las Talitas city, Tucumán province, Argentina [4]. These Brazilian and Argentinean genotypes are often used as parents in each country’s respective breeding programs to produce new cultivars. Due to its importance as a parent in crosses, the genotypes evaluated in this study are potential candidates for germplasm exchange between breeding programs.

The stalks of the accessions were collected, and total genomic DNA was extracted from a fresh meristem cylinder by using the CTAB method [17]. Two independent DNA extractions were performed per sample. DNA concentration was measured with a spectrophotometer, and DNA quality was assessed by running the samples in agarose gel (0.7%) and staining with gelred.

### TRAP marker amplification

Sugarcane DNA samples were characterized by ten combinations of TRAP primers. To compose TRAP markers, three arbitrary (reverse) and ten fixed (forward) primers were used. The arbitrary reverse primers were obtained from [6]. The seven fixed primers associated with sucrose metabolism genes (dirigent protein (DirH6), pyruvate orthophosphate dikinase 3 (PODK3), sucrose phosphate synthase (SuPS), sucrose phosphate synthase 1 (SuPS1), sugar transporter (Sut), sugar transporter 4 (Sut4), and soluble acid invertase 4 (SAI4)), and the one involved with drought tolerance response (dehydration binding factor (DBF)) were based on Alwala et al. [18] and Creste et al. [19], whereas the other two fixed primers associated with lignin metabolism genes (caffeic acid O-methyltransferase (COMT) and ferulate-5-hydroxylase (F5H)) were based on Suman et al. [7].

The amplification reaction mix, optimized in EEAOC´s laboratory, contained: 1x buffer Taq DNA polymerase, 2.5 mM MgCl_2_, 0.75 U Taq DNA polymerase (ThermoFisher), 0.16 μM of both primers, 0.088 mM of each dATP, dTTP and dGTP, 0.072 mM dCTP, 0.8 μM Cy5.5-dCTP (GE Healthcare Life Sciences) and 100 ng DNA. The amplification parameters when PODK3, SuPS and SAI4 fixed primers were used were as follows: one cycle at 94°C for 4 min; 35 cycles at 94°C for 45 s, 45°C for 45 s and 72°C for 1 min; and one cycle at 72°C for 7 min. Amplification parameters when DirH6, DBF, SuPS1, Sut, Sut4, COMT and F5H fixed primers were used were as follows: one cycle at 94°C for 4 min; 5 cycles at 94°C for 45 s, 35°C for 45 s and 72°C for 1 min; 35 cycles at 94°C for 45 s, 50°C for 45 s and 72°C for 1 min and one cycle at 72°C for 7 min.

Amplification products were separated by electrophoresis on denaturing polyacrylamide gels in a 4300 DNA Analyzer (LI-COR). Gels were analyzed by using the SagaMX AFLP Software (LI-COR) and the fragments were scored as ‘‘1” for presence and ‘‘0” for absence to construct a binary matrix. Only clearly distinguishable fragments were scored.

### SSR markers

Analyses of SSR and disease-associated markers were conducted at UFSCar/RIDESA. The SSR markers were amplified based on the procedures described by Oliveira et al. [20], and the amplified fragments were visualized as described by Creste et al. [21]. A total of 10 SSR primers were used [22–25], out of which eight were from expressed sequences (EST-SSR) and two from genomic origin. Due to the polyploid and complex nature of sugarcane, the amplified SSR fragments were evaluated as dominant markers [26]. Therefore, the fragments were classified as binary, i.e., (1) indicated a fragment was present, and (0) indicated a fragment was absent. The polyacrylamide gels were manually evaluated, and a binary matrix was constructed with a combination of the detected fragments.

### Markers associated to disease resistance

All genotypes were amplified to evaluate the presence of *Bru1* [11] and G1 [15] diagnostic markers, associated with brown and orange rust resistance, respectively.

The presence of the *Bru1* gene was detected using R12H16 and 9O20-F4-RsaI molecular markers. PCR conditions and interpretation of results (haplotype 1 to 4) were carried out as proposed by Costet et al. [11].

For the G1 marker, PCR amplifications were performed in 20 μl reaction containing 10X PCR buffer (10 mM Tris-HCl, 50 mM KCl), 2.5 mM MgCl_2_, 0.2 mM each dNTP, 1 μM each forward and reverse primer, 0.5 U Taq DNA polymerase (Promega), 40 ng template DNA and ultrapure water to complete volume. Touchdown PCR was performed, as proposed by Yang et al. [15]. Considering the genotyping results, the cultivars with the presence of the G1 marker, a fragment of approximately 950 base pairs, were encoded with ‘P’, and cultivars with an absence of this fragment were encoded with ‘A’.

### Molecular data analysis

Polymorphic locus proportion (PLP95) and the polymorphism information content (PIC) [27] were calculated for SSR and TRAP markers by using the *Info-Gen* software [28]. The former considers a locus as polymorphic if it has population variations and the most common allele frequency does not exceed 95%, whereas PIC is frequently used to determine the marker value in detecting polymorphism.

Besides, the number of total polymorphic bands, Heterozygosity observed (Ho), Heterozygosity expected (He) and Nei’s diversity index were calculated by using GenAlEx v 6.5 [29] for each primer combination and population.

Genetic structure was determined through genetic similarity, analysis of molecular variance (AMOVA), Multidimensional scaling (MDS), and Bayesian method employing Structure software by using molecular data from TRAP and SSR, together and separately.

Similarity was calculated by using Jaccard coefficient (Sj) [30]. Cluster analyses were carried out using Ward method and distances expressed as 1—Sj. All calculations were carried out by using *Info-Gen* software with its interface in R.

Mantel correlations among distance matrices obtained by each trait by TRAP, and by SSR, AMOVA and MDS were calculated by using *Info-Gen* software, and population structure to verify the number of subpopulations by Structure software [31]. AMOVA was performed considering two different origins related to the two different breeding programs, RIDESA (49 genotypes) and EEAOC (47 genotypes) from Brazil and Argentina, respectively, that the genotypes belong to.

## Results

### Genetic diversity and population structure

A total of 436 TRAP fragments were identified, out of which 387 (88.7%) were polymorphic among the evaluated sugarcane genotypes. Respecting SSR, a total of 138 fragments were amplified, being 93% polymorphic (S2 Table). It was possible to discriminate each genotype with both TRAP and SSR markers.

Polymorphic locus proportion (PLP(95)) values ranged between 0.57 and 0.98 for each combination of TRAP primers and between 0.61 and 1 for each SSR primer. PIC values ranged between 0.18 and 0.32 for TRAP primers whereas for SSR primers ranged from 0.24 to 0.32. Average Ho values were 0.24 and 0.28, whereas average He values were 0.25 and 0.28, for TRAP and SSR primers, respectively (S2 Table). Regarding diversity indexes calculated for each population, they revealed similar values (S3 Table).

Mantel correlations among distance matrices were low to moderate and most of them statistically significant. The highest value was obtained between lignin and sucrose for TRAP markers (0.42; p < 0.0001), whereas the lowest significant value was obtained between sucrose TRAP markers and SSR (0.19; p < 0.0001) (Table 1). For all analyses, two main groups were clearly obtained.

**Table 1:** Mantel correlations among TRAP and SSR distance matrices.

		TRAP		SSR
		Sucrose	Lignin	
**TRAP**	**Drought tolerance**	0.29 (p = 0.0001)	0.33 (p < 0.0001)	0.08 (p < 0.066)
	**Sucrose**		0.42 (p < 0.0001)	0.19 (p < 0.0001)
	**Lignin**			0.20 (p < 0.0001)
	**All traits together**			0.22 (p < 0.0001)

Although the correlation between SSR and TRAP matrixes was relatively low (0.22: p < 0.001), only 13 were assigned to different groups by TRAP and SSR analysis (S1 Table). The three from EEAOC´s germplasm bank (HOCP91-555, HOCP92-624 and TUC96-46) belong to group 1 by TRAP markers; however, they belong to group 2 when SSR data was analysed alone. In a similar way, the ten from RIDESA´s germplasm bank (RB855036, RB855589, RB867515, RB92579, RB975242, RB975952, SP80-1520, SP80-1816, SP80-185 and SP80-3280) belong to group 2 by SSR; however, when TRAP data was analysed alone they belong to group 1.

**Fig 1:**
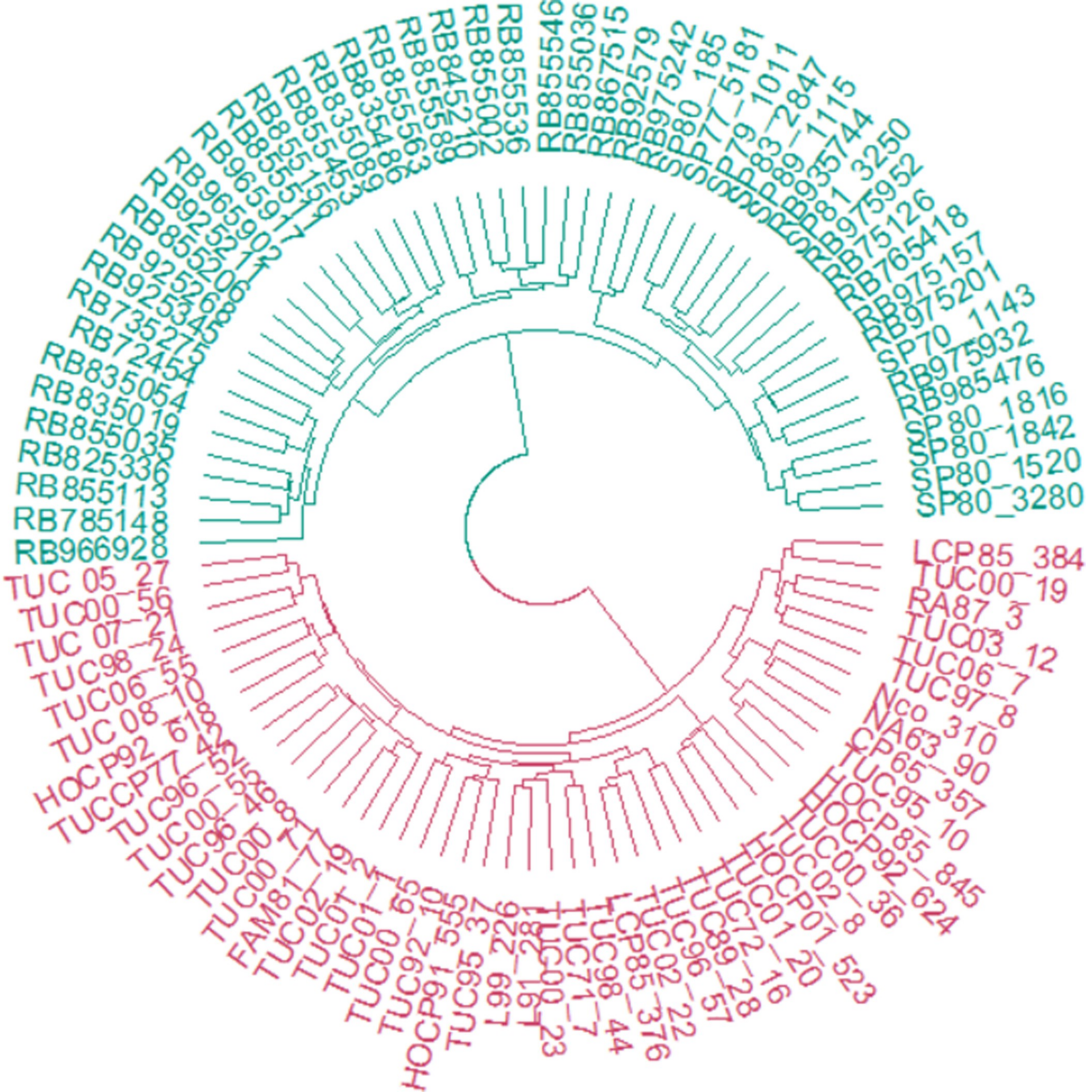
Genetic diversity dendrogram of 96 sugarcane varieties calculated with TRAP and SSR data by using Jaccard coefficient and Ward clustering method in R software. The different colours correspond to the two main groups obtained, representing the two breeding programs.

According to the circular dendrogram obtained by using TRAP and SSR data together, genotypes were clustered in two main groups (Fig 1 and S1 Table). The first one (1) grouped Argentinean genotypes (TUC, NA, RA and FAM varieties) and American genotypes (CP, HoCP, L, LCP) closely related to Argentinean ones since USA is the main source of germplasm for EEAOC breeding program. The second group (2) clustered all the RB and SP varieties.

When analysing the genetic structure through Structure software, also two subpopulations were identified (k = 2) (S1 Table). Results obtained by using TRAP and SSR data together for genetic diversity analysis and structure revealing the same clusters and the 13 genotypes aforementioned belong to the expected group.

AMOVA was performed considering two different origins related to the two different breeding programs, calculated by using TRAP and SSR information, together and separately (Table 2). For all situations, the percentage of variation was greater inside the breeding program than between them (p <0.0001). In addition, the F_ST_ values indicate a moderate genetic differentiation between the two breeding programs.

**Table 2:** Analysis of molecular variance (AMOVA) between and within Brazilian and Argentinean breeding programs.

Molecular marker	V.S.	d.f.	S.S.	Variance components	% variation	Fixation index (F_ST_)	*P* value
**TRAP**	Between breeding programs	1	493.72	9.56	14.56	0.15	<0.0001
	Within breeding programs	90	5044.55	56.05	85.44		
**SSR**	Between breeding programs	1	142.15	2.63	11.51	0.12	<0.0001
	Within breeding programs	91	1840.34	20.22	88.49		
**TRAP + SSR**	Between breeding programs	1	623.38	12.26	13.85	0.14	<0.0001
	Within breeding programs	88	6708.67	76.23	86.15		

**V.S.**: variation source; **d.f.**: degree freedom, **S.S.**: sum square.

When MDS was performed, the main exes explained 18.6% of the variability expressed among genotypes (S1 Fig). The arrangement of genotypes was similar to that previously found by cluster and Structure analysis since they were grouped according to their origin and were genetically diverse.

### Markers associated to disease resistance

**Fig 2:**
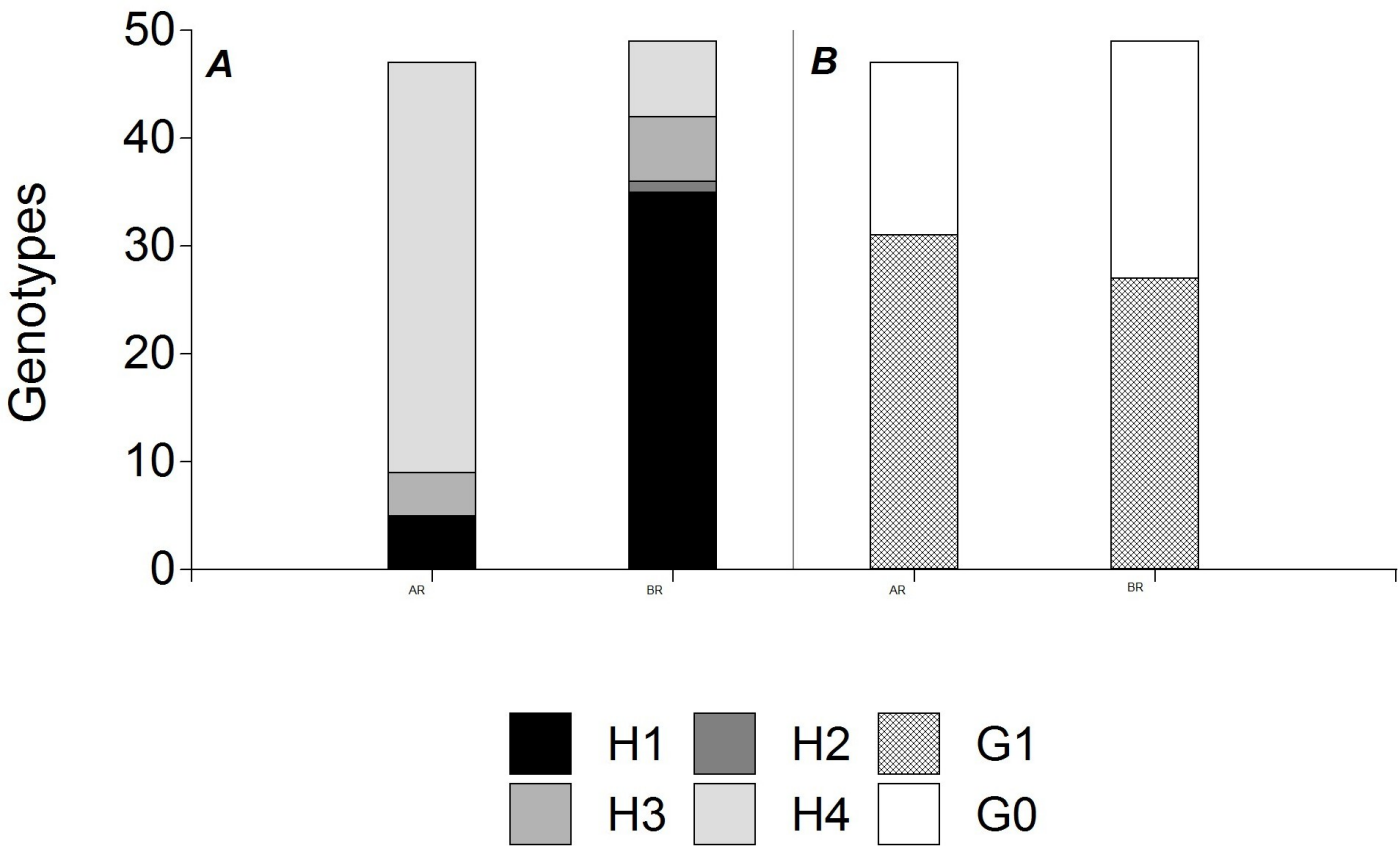
Presence of markers associated with disease resistance in Argentinean and Brazilian genotypes. **A** *Bru1* haplotypes associated with brown rust resistance (H1: presence of both diagnostic markers, H2 and H3: presence of only one associated marker and H4: absence of both markers). **B** G1 marker associated with orange rust resistance (G1: presence and G0: absence).

The results on the *Bru*1 markers are shown in Fig 2A. Considering all genotypes, the percentage of haplotypes 1 and 4 was 41.74% and 46.60%, respectively. Brazilian genotypes showed a higher frequency of the haplotype 1 associated with the presence of both markers (72%). On the other hand, the genotypes from Argentina had a low frequency of haplotype 1 (8%), and a high frequency of haplotype 4 (absence of both markers, 82%).

Most of the evaluated genotypes (58.25%) showed the presence of the fragment associated with the G1 marker (Fig 2B). The Brazilian accessions showed 28 genotypes with the presence of G1 and 22 sugarcane clones with the absence of this marker. For the Argentine germplasm, 66.66% of the accessions showed the presence of the G1 marker.

## Discussion

A better understanding of the genetic diversity in the germplasm available for breeding is a necessary first step to decide how to compartmentalize the pattern of variation found in the collection and to select potential parents for crossing aimed at efficient introgression of desirable alleles in order to fulfill breeding objectives [7]. For that sugarcane breeding programs must have a diverse germplasm bank whose genetic diversity and population structure must be well characterized. In that sense, the molecular profile is more useful than phenotypic traits influenced by environmental effects, to characterize in a more reliable way accessions.

TRAP markers anchored in sucrose and lignin metabolism and drought tolerance genes and SSR markers were able to detect genetic differentiation within and between EEAOC and RIDESA breeding programs from Argentina and Brazil, respectively. This suggested that even for these genes under selection process) [1], considering not only those where TRAP markers were anchored but also the eight SSR associated with expressed sequences (EST-SSR), there is still possibility of introgression of new alleles, not only from different breeding programs but also within them. It is well known that yield gain rates are decreasing over time in sugarcane, suggesting a possible plateau in sugar accumulation [32], for that new sugarcane breeding strategies, including the use of molecular markers to better characterize parents, are essential to overcome this limitation [33].

In the evaluated genotypes a higher number of fragments was amplified with TRAP markers than with SSR; however, a little higher percentage of polymorphism was detected with SSR than with TRAP markers. Respect to PIC value, it is often used to measure the discriminatory power of a genetic marker system and its theoretical maximum value for a dominant marker is 0.5) [34]. The most discriminatory TRAP maker was SAI4/Arbi1 (PIC: 0.32); whereas for SSR, the SSR 01 was the highest PIC value (0.32). In a previous study conducted on 36 parents of the EEAOC sugarcane breeding program characterized with 15 SSR primers, PIC values ranged in similar values (0.18 to 0.32, average: 0.26) [10]; however, only nine bands were obtained on average for each SSR primer. In the case of TRAP markers, those proposed by Alwala et al. [18], Creste et al. [19] and Suman et al. [7] were used in the present work. In the first study, PIC values ranged from 0.11 to 0.36 (average: 0.24), in the second ranged from 0.18 to 0.42 (average: 0.30) and in the last one from 0.20 to 0.35 (average: 0.28). The highest values could be related to the diversity sampled since in Alwala et al. [18], three genera were characterized (*Saccharum*, *Miscanthus*, and *Erianthus*) while in Creste et al. [19] and Sunman et al. [7], two (*Saccharum* and *Erianthus*). In Alwala et al. [18] the average number of bands obtained considered six TRAP markers (five primers involved in sucrose metabolism and one in cold tolerance) was 33.33, whereas in Creste et al. [19], 16.5 bands were obtained on average by amplifying 30 primers (21 anchored to sucrose metabolism and nine to drought response genes). The higher number obtained in this study could be related to both the detection method (silver staining vs. Li-cor system) and the genes involved since in Suman et al. [7], 58.5 bands were obtained by employing 16 lignin gene-based TRAP primers. Similarly in Medeiros et al. [1], an average of 74.37 fragments per locus were obtained when six TRAP primers based on sucrose metabolism and two on lignin metabolism were employed.

When Mantel correlations were performed among distance matrices for each kind of molecular marker/trait, values obtained were low to moderate but most of them statistically significant. Interestingly the highest value was obtained between lignin and sucrose for TRAP markers. This means that there is a correlation between the genetic distances among the genotypes calculated by using sucrose and lignin TRAP markers. Historically, to increase sugar content has been one of the main goals of sugarcane breeding programs around the world whereas decreasing lignin content to facilitate cellulose saccharification for second-generation ethanol production for energy cane proposes it is a recently incorporated aim to sugarcane breeding programs [1]. Considering that sugarcane is one of the most important grass biofuel crops due to its ability to produce high biomass, the development of genotypes with high biomass yield and low or modified lignin is now a major objective of breeders [7]. For that, the genetic variation associated with lignin genes will be useful to design breeding strategies to develop superior energy cane genotypes. A further study would be to associate the genetic variation found with phenotypic variations for lignin content in the evaluated sugarcane genotypes.

On the other hand, the lowest Mantel´s significant correlation value was obtained between sucrose TRAP markers and SSR. Similarly, a low correlation was observed between TRAP markers and SSR, probably due to most TRAP markers were anchored to sucrose metabolism.

Genetic similarity analysis, Structure and MDS revealed two groups clearly differentiated, associated with the two breeding programs. Out of the 96 genotypes, 13 were assigned to different groups by TRAP and SSR analysis. However, when SSR and TRAP marker data were analysed together, it better reflected the breeding program that genotypes belong to. Both RIDESA and EEAOC sugarcane breeding programs, have as their main objective the development of improved varieties with high cane yield, high sucrose content, disease resistance, rapid sprouting and ratoon ability [2, 4]. Both have been equally efficient in producing cultivars with high sugar levels in their selection process but adapted to their specific agro-ecological conditions. In order to perform the crossing, EEAOC breeding program employed as parents clones selected from advanced stage trials in a recurrent selection process, and commercially grown cultivars where materials from USA programs constitute the main source of foreign parents (L, CP, Ho) [4], whereas in the case of the RIDESA program, it employed several RB and SP varieties (S1 Table). So, the differentiation between the genotypes of each breeding program can be explained considering that the allele pool is different in each germplasm bank. Besides, it should be taken into account that the environments where genotypes were selected are very different (Tucumán is located in a subtropical region and sugarcane generally does not flower there naturally whereas Brazil is located in a tropical region), and as GE interaction substantially influences cultivar performance [33], results of the present work suggested that despite selection and evaluation methods are similar in both breeding programs, the frequency of alleles that governed traits under selection could be very different. EEAOC and RIDESA programs are conducted in regions that are separated by latitude (sugarcane area in Tucumán where selection localities of the EEAOC breeding program are included is located between 26°21´24´´ S and 27°56´25´´ S [4], whereas in Brazil is between 7°16’27.4"S and 26°42’49.3"S) as in the case of the selection programs conducted within six different regions in Australia that have the characteristics of mega-environments [35]. In agreement with this hypothesis, when nutritional component traits were evaluated in cultivars from different breeding programs and genetic backgrounds, those from locations with similar geographical and climate conditions revealed high similarity [33].

AMOVA revealed more variability within breeding programs than between them. In a similar way, molecular variance found by TRAP markers was higher within populations than among populations even considering ancestor accessions, hybrids from Brazilian breeding programs and hybrids from other breeding programs [1]. Also when Glynn et al. [36] compared sugarcane clones from three different mainland USA growing areas, 96.6% of variation within populations was detected. Besides results obtained by Tazeb et al. [37] after characterizing genotypes introduced to Ethiopia from Barbados, Cuba, France, India, South Africa, Sudan, and USA revealed more variability within groups than among them. Even when *S*. *officinarum* and *S*. *spontaneum* genotypes were compared similar results were found [38]. This result suggested that even inside each breeding program there is still a broad genetic background and allelic pool to be explored. It must be noticed that although genetic similarity analysis, Structure and MDS clearly revealed two groups, by AMOVA the genetic differentiation between the two breeding programs was moderate. This could be associated with the fact that genetic diversity indexes for each population (Ho, He and Nei) were very similar.

Molecular markers are valuable biotechnological tools to assist conventional breeding not only to estimate genetic diversity and select the best parental combinations for crossings, but also to improve the precision and efficiency of selection at each stage. As it was mentioned before, the most effective method to control several sugarcane diseases is the use of resistant commercial varieties, where molecular markers linked to resistance genes that explain enough percentage of the phenotypic variation observed, could be of great help to predict a resistant behaviour. Besides, genotypes harbouring resistant genes could be used as parents in crossing to assure a resistant progeny. In this paper, the prevalence of *Bru1* gene related to brown rust resistance within the genetic base was estimated by determining its frequency among 96 genotypes. The low prevalence of the *Bru1* gene in the EEAOC´s germplasm was previously reported by Racedo et al. [12], whereas, Brazilian accessions showed a high frequency of haplotype I, revealing a genetic fixation effect of *Bru1* in this germplasm, mainly for breeding genotypes. Similar results were presented by Neuber et al. [14] and Barreto et al. [39].

Regarding the G1 molecular marker, associated with the orange rust resistance gene, the results obtained in this paper for Brazilian accessions were similar to those obtained by Fier et al. [16]. This is the first report of the use of marker G1 to identify resistance to orange rust, in sugarcane germplasm from Argentina. Although *P*. *kuehnii* was first reported in Argentina in 2015 [40] and fortunately it is still confined to the site where it was detected in the north east of the country, the information obtained is very useful in case the disease arrives in Tucumán, the main sugarcane producer of the country.

In summary, the present work contributed to estimate genetic diversity within and between two breeding programs, revealing that both constitute two groups, genetically diverse. This aspect reinforces the importance of germplasm exchange between breeding programs in order to expand the genetic base of breeders’ working collections. Besides, the use of molecular tools could allow improving the assertiveness of the crosses and efficiency of introgression of favourable alleles related to sucrose and lignin synthesis and disease resistance, increasing the probability of obtaining more productive and/or resistant varieties.

## Supporting information

S1 Fig Multidimensional scaling of 96 sugarcane varieties based on TRAP and SSR markers. The different colors indicate the breeding programs that the genotypes belong to.
(JPG)

S1 Table Sugarcane genotypes, their pedigree information, origin and groups obtained by dendrogram and Structure analysis. (DOCX)

S2 Table Total and polymorphic bands obtained for each combination of TRAP and SSR primers tested. (DOCX)

S3 Table Diversity indexes for each population. (DOCX)
