# Supplementary figures and images for: Genetic diversity and population structure of Saccharum hybrids
Source: PLoS One. 2023 Aug 15;18(8):e0289504. doi: 10.1371/journal.pone.0289504 (PMC10426985; doi:10.1371/journal.pone.0289504)

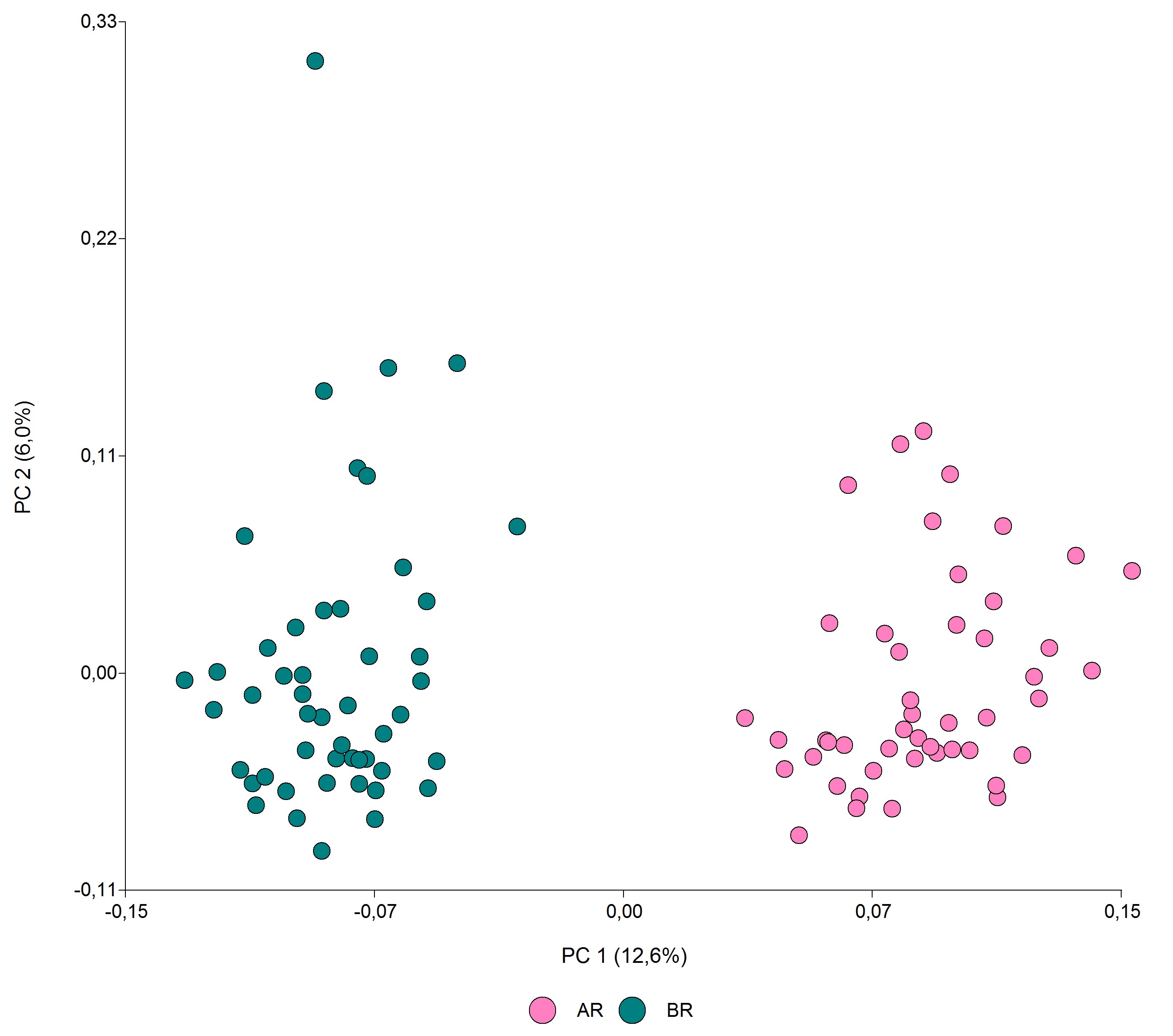

Supplement: S1 Fig — The different colors indicate the breeding programs that the genotypes belong to. (JPG) [file pone.0289504.s001.jpg]
