# Supplementary material for: Genetic diversity and population structure of Saccharum hybrids
Source: PLoS One. 2023 Aug 15;18(8):e0289504. doi: 10.1371/journal.pone.0289504 (PMC10426985; doi:10.1371/journal.pone.0289504)
Supplement: S1 Table — (DOCX) [file pone.0289504.s002.docx]

**S1 Table.** Sugarcane genotypes, their pedigree information, origin and groups obtained by dendrogram and Structure analysis.

| **Genotype** | **Grandparents** ♀ | | **Grandparents** ♂ | | **Parents** | | **Origin** | **Code** | **Group** |
| --- | --- | --- | --- | --- | --- | --- | --- | --- | --- |
|  | **Female** | **Male** | **Female** | **Male** | **Female** | **Male** |  |  |  |
| CP65-357 | Na | Na | Na | Na | CP52-68 | CP53-17 | Canal Point (USA) | AR | 1 |
| FAM81-77 | Na | Na | Na | Na | Na | Na | Tucumán (Argentina) | AR | 1 |
| HOCP01-523 | Na | Na | Na | Na | Na | Na | Houma-Canal Point (USA) | AR | 1 |
| HOCP85-845 | CP61-037 | CP52-068 | Na | Na | CP72-370 | CP77-403 | Houma-Canal Point (USA) | AR | 1 |
| HOCP91-555 | Na | Na | Na | Na | CP83-644 | LCP82-94 | Houma-Canal Point (USA) | AR | 1 (2) |
| HOCP92-618 | Na | Na | Na | Na | CP78-304 | LCP81-30 | Houma-Canal Point (USA) | AR | 1 |
| HOCP92-624 | Na | Na | Na | Na | CP81-325 | CP71-1038 | Houma-Canal Point (USA) | AR | 1 (2) |
| L91-281 | Na | Na | Na | Na | CP78-317 | LCP81-30 | Louisiana (USA) | AR | 1 |
| L99-226 | Na | Na | Na | Na | HOCP 89-846 | LCP81-30 | Louisiana (USA) | AR | 1 |
| LCP85-376 | Na | Na | Na | Na | CP77-403 | Polycross | Louisiana-Canal Point (USA) | AR | 1 |
| LCP85-384 | Na | Na | Na | Na | CP77-310 | CP77-407 | Louisiana-Canal Point (USA) | AR | 1 |
| NA63-90 | Na | Na | Co421 | Co312 | CP52-68 | NCO 310 | Norte Argentino (Argentina) | AR | 1 |
| NCO-310 | Na | Na | Na | Na | Co421 | Co312 | South Africa | AR | 1 |
| RA87-3 | Na | Na | CL47-143 | CP53-017 | TUC5-25 | CP57-614 | Tucumán (Argentina) | AR | 1 |
| RB72454 | F36-819 | CP36-46 | Na | Na | CP53-76 | Na | RIDESA (Brazil) | BR | 2 |
| RB735275 | CP27-108 | Na | Na | Na | IAC49-131 | Na | RIDESA (Brazil) | BR | 2 |
| RB75126 | Badila | 28 NG 251 | Na | Na | C278 | Na | RIDESA (Brazil) | BR | 2 |
| RB765418 | B34104 | M213/40 | Na | Na | M253/48 | Na | RIDESA (Brazil) | BR | 2 |
| RB785148 | Co419 | Na | Na | Na | IAC47-31 | Na | RIDESA (Brazil) | BR | 2 |
| RB825336 | H48-3717 | Na | Na | Na | H53-3989 | Na | RIDESA (Brazil) | BR | 2 |
| RB835019 | CP53-76 | Na | Co419 | Co419 | RB72454 | NA56-79 | RIDESA (Brazil) | BR | 2 |
| RB835054 | CP53-76 | Na | Co419 | Co419 | RB72454 | NA56-79 | RIDESA (Brazil) | BR | 2 |
| RB835089 | CP53-76 | Na | Co419 | Co419 | RB72454 | NA56-79 | RIDESA (Brazil) | BR | 2 |
| RB835486 | CP52-1 | CP48-103 | Na | Na | L60-14 | Na | RIDESA (Brazil) | BR | 2 |
| RB845210 | CP53-76 | Na | IAC48/65 | Na | RB72454 | SP70-1143 | RIDESA (Brazil) | BR | 2 |
| RB855002 | IAC48/65 | Na | CP53-76 | Na | SP70-1143 | RB72454 | RIDESA (Brazil) | BR | 2 |
| RB855035 | CP52-1 | CP48-103 | CB41-76 | Na | L60-14 | SP70-1284 | RIDESA (Brazil) | BR | 2 |
| RB855036 | CP53-76 | Na | IAC48/65 | Na | RB72454 | SP70-1143 | RIDESA (Brazil) | BR | 2 (1) |
| RB855113 | IAC48/65 | Na | CP53-76 | Na | SP70-1143 | RB72454 | RIDESA (Brazil) | BR | 2 |
| RB855156 | CP53-76 | Na | CP52-68 | CP62-258 | RB72454 | TUC71-7 | RIDESA (Brazil) | BR | 2 |
| RB855206 | CP53-76 | Na | CP52-68 | CP62-258 | RB72454 | TUC71-7 | RIDESA (Brazil) | BR | 2 |
| RB855453 | CP52-68 | CP62-258 | Na | Na | TUC71-7 | Na | RIDESA (Brazil) | BR | 2 |
| RB855511 | NA56-79 | Na | Na | Na | SP71-1406 | Na | RIDESA (Brazil) | BR | 2 |
| RB855536 | IAC48/65 | Na | CP53-76 | Na | SP70-1143 | RB72454 | RIDESA (Brazil) | BR | 2 |
| RB855546 | IAC48/65 | Na | CP53-76 | Na | SP70-1143 | RB72454 | RIDESA (Brazil) | BR | 2 |
| RB855563 | CP52-68 | CP62-258 | IAC48/65 | Na | TUC71-7 | SP70-1143 | RIDESA (Brazil) | BR | 2 |
| RB855589 | IAC48/65 | Na | CP52-68 | CP62-258 | SP70-1143 | TUC71-7 | RIDESA (Brazil) | BR | 2 (1) |
| RB867515 | CP53-76 | Na | Na | Na | RB72454 | Na | RIDESA (Brazil) | BR | 2 (1) |
| RB925211 | RB72454 | TUC71-7 | Na | Na | RB855206 | Na | RIDESA (Brazil) | BR | 2 |
| RB925268 | SP71-1406 | Na | Na | Na | RB855511 | Na | RIDESA (Brazil) | BR | 2 |
| RB925345 | H50-676 | H49-3646 | Na | Na | H59-1966 | Na | RIDESA (Brazil) | BR | 2 |
| RB92579 | C278 | Na | NCo334 | Na | RB75126 | RB72199 | RIDESA (Brazil) | BR | 2 (1) |
| RB935744 | RB72454 | NA56-79 | M253/48 | Na | RB835089 | RB765418 | RIDESA (Brazil) | BR | 2 |
| RB965902 | SP70-1143 | RB72454 | TUC71-7 | Na | RB855536 | RB855453 | RIDESA (Brazil) | BR | 2 |
| RB965917 | TUC71-7 | Na | SP70-1143 | RB72454 | RB855453 | RB855536 | RIDESA (Brazil) | BR | 2 |
| RB966928 | RB72454 | TUC71-7 | IAC49/131 | NA56-79 | RB855156 | RB815690 | RIDESA (Brazil) | BR | 2 |
| RB975157 | TUC71-7 | SP70-1143 | Co331 | Na | RB855563 | RB735200 | RIDESA (Brazil) | BR | 2 |
| RB975201 | SP70-1143 | RB72454 | Na | Na | RB855113 | Na | RIDESA (Brazil) | BR | 2 |
| RB975242 | H32-8560 | PT43-52 | Na | Na | F147 | Na | RIDESA (Brazil) | BR | 2 (1) |
| RB975932 | BO17 | IAC50/134 | RB72454 | TUC71-7 | SP80-185 | RB855206 | RIDESA (Brazil) | BR | 2 |
| RB975952 | L60-14 | Na | F150 | Na | RB835486 | RB825548 | RIDESA (Brazil) | BR | 2 (1) |
| RB985476 | H48-3717 | Na | RB72454 | TUC71-7 | H53-3989 | RB855206 | RIDESA (Brazil) | BR | 2 |
| SP70-1143 | CP27-108 | Na | Na | Na | IAC48-65 | Na | COPERSUCAR (Brazil) | BR | 2 |
| SP77-5181 | H40-1184 | Na | Na | Na | HJ57-41 | Na | COPERSUCAR (Brazil) | BR | 2 |
| SP79-1011 | Co419 | Co419 | Na | Na | NA56-79 | Co775 | COPERSUCAR (Brazil) | BR | 2 |
| SP80-1520 | H40-779 | Na | CP5530 | CP5376 | H48-3166 | SP71-1088 | COPERSUCAR (Brazil) | BR | 2 (1) |
| SP80-1816 | CP5530 | CP5376 | H49134 | Na | SP71-1088 | H57-5028 | COPERSUCAR (Brazil) | BR | 2 (1) |
| SP80-1842 | CP5530 | CP5376 | H49134 | Na | SP71-1088 | H57-5028 | COPERSUCAR (Brazil) | BR | 2 |
| SP80-185 | Co331 | Co326 | Na | Na | BO17 | Na | COPERSUCAR (Brazil) | BR | 2 (1) |
| SP80-3280 | CP5530 | CP5376 | H49134 | Na | SP71-1088 | H57-5028 | COPERSUCAR (Brazil) | BR | 2 (1) |
| SP81-3250 | CP62-374 | CP57-526 | CB49-260 | Na | CP70-1547 | SP71-1279 | COPERSUCAR (Brazil) | BR | 2 |
| SP83-2847 | H40-1184 | Na | IAC48/65 | Na | HJ5741 | SP70-1143 | COPERSUCAR (Brazil) | BR | 2 |
| SP89-1115 | CP66-1043 | CP56-63 | Na | Na | CP73-1547 | Na | COPERSUCAR (Brazil) | BR | 2 |
| TUC 05-27 | CP81-325 | CP71-1038 | CP71-321 | US72-19 | HOCP92-631 | TUC72-16 | Tucumán (Argentina) | AR | 1 |
| TUC 07-21 | CP83-644 | LCP82-94 | TUC83-1 | TUC77-37 | HOCP91-555 | TUC92-10 | Tucumán (Argentina) | AR | 1 |
| TUC 08-10 | CP77-310 | CP77-407 | CP65-357 | S87-1756 | LCP85-384 | TUC95-37 | Tucumán (Argentina) | AR | 1 |
| TUC 97-8 | TUC77-006 | TUC67-027 | CP71-321 | US72-19 | TUC87-21 | TUCCP77-42 | Tucumán (Argentina) | AR | 1 |
| TUC00-19 | CP83-644 | CP70-321 | CP71-321 | US72-19 | HOCP92-675 | TUCCP77-42 | Tucumán (Argentina) | AR | 1 |
| TUC00-23 | CP81-325 | CP71-1038 | CP71-321 | US72-19 | HOCP92-631 | TUC72-16 | Tucumán (Argentina) | AR | 1 |
| TUC00-36 | CP77-310 | CP77-407 | CP77-310 | CP77-407 | LCP85-384 | LCP85-384 | Tucumán (Argentina) | AR | 1 |
| TUC00-55 | CP81-325 | CP71-1038 | TUC77-003 | TUC77-004 | HOCP92-631 | TUC86-21 | Tucumán (Argentina) | AR | 1 |
| TUC00-56 | CP81-325 | CP71-1038 | TUC77-003 | TUC77-004 | HOCP92-631 | TUC86-21 | Tucumán (Argentina) | AR | 1 |
| TUC00-65 | CP83-644 | LCP82-94 | Na | Na | HOCP91-555 | Polycross | Tucumán (Argentina) | AR | 1 |
| TUC00-71 | Na | Na | Na | Na | Na | Na | Tucumán (Argentina) | AR | 1 |
| TUC00-8 | CP83-644 | CP70-321 | CP71-321 | US72-19 | HOCP92-675 | TUCCP77-42 | Tucumán (Argentina) | AR | 1 |
| TUC01-1 | CP81-325 | CP71-1038 | Na | Na | HOCP92-631 | TUC77-16 | Tucumán (Argentina) | AR | 1 |
| TUC01-2 | CP81-325 | CP71-1038 | Na | Na | HOCP92-631 | TUC77-16 | Tucumán (Argentina) | AR | 1 |
| TUC01-20 | CP83-644 | LCP82-94 | TUC74-027 | S75-251 | HOCP91-555 | TUC84-31 | Tucumán (Argentina) | AR | 1 |
| TUC02-19 | CP77-310 | CP77-407 | CP84-722 | LCP81-30 | LCP85-384 | HOCP93-750 | Tucumán (Argentina) | AR | 1 |
| TUC02-22 | Na | Na | Na | Na | Na | Na | Tucumán (Argentina) | AR | 1 |
| TUC02-8 | Na | Na | Na | Na | Na | Na | Tucumán (Argentina) | AR | 1 |
| TUC03-12 | CP81-325 | CP71-1038 | CP71-321 | US72-19 | HOCP92-631 | TUC72-16 | Tucumán (Argentina) | AR | 1 |
| TUC06-55 | TUC80-002 | TUC79-009 | LCP81-10 | CP72-356 | TUC93-98 | HOCP91-552 | Tucumán (Argentina) | AR | 1 |
| TUC06-7 | TUC78-009 | TUC77-042 | CP77-310 | CP77-407 | TUC89-32 | LCP85-384 | Tucumán (Argentina) | AR | 1 |
| TUC71-7 | Na | Na | Na | Na | CP52-68 | CP62-258 | Tucumán (Argentina) | AR | 1 |
| TUC72-16 | Na | Na | Na | Na | CP71-321 | US72-19 | Tucumán (Argentina) | AR | 1 |
| TUC89-28 | CP71-321 | US72-19 | CP71-321 | US72-19 | TUCCP77-42 | TUCCP77-42 | Tucumán (Argentina) | AR | 1 |
| TUC92-10 | Na | Na | Na | Na | TUC83-1 | TUC77-37 | Tucumán (Argentina) | AR | 1 |
| TUC95-10 | CP61-037 | CP52-068 | CL47-143 | CP53-017 | CP72-370 | CP57-614 | Tucumán (Argentina) | AR | 1 |
| TUC95-37 | CP52-68 | CP53-17 | TUC72-16 | CP57-614 | CP65-357 | S87-1756 | Tucumán (Argentina) | AR | 1 |
| TUC96-46 | NA63-090 | TUC74-015 | TUC74-010 | TUC68-019 | TUC84-20 | TUC84-24 | Tucumán (Argentina) | AR | 1 (2) |
| TUC96-52 | CP77-310 | CP77-407 | TUC74-045 | NA56-079 | LCP85-384 | TUC83-8 | Tucumán (Argentina) | AR | 1 |
| TUC96-57 | Na | Na | Na | Na | Na | Na | Tucumán (Argentina) | AR | 1 |
| TUC98-24 | CP67-411 | Polycross | TUC77-006 | TUC67-027 | TUC79-9 | TUC87-21 | Tucumán (Argentina) | AR | 1 |
| TUC98-44 | CP81-325 | CP71-1038 | TUC72-025 | CP57-614 | HOCP92-631 | S87-419 | Tucumán (Argentina) | AR | 1 |
| TUCCP77-42 | Na | Na | Na | Na | CP71-321 | US72-19 | Tucumán - Canal Point (Argentina) | AR | 1 |

Na: not available information. G: group obtained by all diversity (TRAP, SRR and TRAP + SSR) and Structure analysis; in parenthesis the number of the group assigned by only one analysis. Underlie genotypes belong to different groups according to the data analysis.
