# Supplementary material for: Genetic diversity and population structure of Saccharum hybrids
Source: PLoS One. 2023 Aug 15;18(8):e0289504. doi: 10.1371/journal.pone.0289504 (PMC10426985; doi:10.1371/journal.pone.0289504)
Supplement: S2 Table — (DOCX) [file pone.0289504.s003.docx]

**S2 Table**. Total and polymorphic bands obtained for each combination of TRAP and SSR primers tested.

| **Marker** | **Trait** | **Primer combination** | **Reference** | **Total bands** | **Polymorphic bands** | **PLP(95)**** | **PIC***** | **Ho****** | **He******* | **Nei genetic diversity** |
| --- | --- | --- | --- | --- | --- | --- | --- | --- | --- | --- |
| TRAP | Drought tolerance | DBF/Arbi1* | Creste et al. [19] | 49 | 46 | 0.78 | 0.24 | 0.25 | 0.26 | 0.38 |
|  | Sucrose | DirH6/Arbi3 | Creste et al. [19] | 34 | 30 | 0.74 | 0.24 | 0.24 | 0.25 | 0.44 |
|  |  | PODK3/Arbi1 | Alwala et al. [18] | 31 | 26 | 0.81 | 0.27 | 0.25 | 0.26 | 0.40 |
|  |  | SuPS/Arbi3 |  | 63 | 60 | 0.57 | 0.18 | 0.18 | 0.18 | 0.30 |
|  |  | SuPs1/Arbi3 | Creste et al. [19] | 28 | 19 | 0.61 | 0.22 | 0.16 | 0.17 | 0.29 |
|  |  | Sut/Arbi2 |  | 38 | 29 | 0.66 | 0.28 | 0.24 | 0.25 | 0.40 |
|  |  | Sut4/Arbi3 |  | 48 | 36 | 0.56 | 0.22 | 0.19 | 0.19 | 0.32 |
|  |  | SAI4/Arbi1 | Alwala et al. [18] | 56 | 56 | 0.98 | 0.32 | 0.38 | 0.39 | 0.47 |
|  | Lignin | COMT/Arbi1 | Suman et al. [7] | 54 | 54 | 0.83 | 0.27 | 0.32 | 0.33 | 0.46 |
|  |  | F5H/Arbi1 |  | 35 | 31 | 0.66 | 0.23 | 0.24 | 0.25 | 0.41 |
| **Total** | | |  | **436** | **387** |  |  |  |  |  |
| **Average/TRAP marker** | | |  | 43.6 | 38.7 | 0.72 | 0.25 | 0.24 | 0.25 | 0.39 |
| SSR | | SSR 01 | Cordeiro et al. [23] | 19 | 19 | 1 | 0.32 | 0.29 | 0.30 | 0.50 |
|  |  | SSR 02 | Maccheroni et al. [24] | 13 | 13 | 0.85 | 0.27 | 0.29 | 0.30 | 0.50 |
|  |  | SSR 03 | Oliveira et al. [22] | 15 | 15 | 1 | 0.26 | 0.30 | 0.30 | 0.49 |
|  |  | SSR 04 |  | 14 | 12 | 0.79 | 0.31 | 0.28 | 0.29 | 0.47 |
|  |  | SSR 05 |  | 8 | 6 | 0.75 | 0.28 | 0.21 | 0.21 | 0.49 |
|  |  | SSR 06 |  | 13 | 10 | 0.69 | 0.24 | 0.38 | 0.38 | 0.48 |
|  |  | SSR 07 | Marconi et al. [25] | 13 | 13 | 1 | 0.25 | 0.34 | 0.34 | 0.50 |
|  |  | SSR 08 |  | 15 | 14 | 0.80 | 0.25 | 0.24 | 0.24 | 0.48 |
|  |  | SSR 09 |  | 13 | 13 | 0.85 | 0.19 | 0.21 | 0.21 | 0.47 |
|  |  | SSR 10 | Oliveira et al. [22] | 15 | 14 | 0.87 | 0.28 | 0.27 | 0.28 | 0.49 |
| **Total** |  |  |  | **138** | **129** |  |  |  |  |  |
| **Average/SSR primer** | |  |  | 13.8 | 12.9 | 0.86 | 0.26 | 0.28 | 0.28 | 0.49 |
| **Total** |  |  |  | **574** | **516** |  |  |  |  |  |

*Arbi primers were obtained from Li and Quiros [5].
**Polymorphic locus proportion where the most common allele frequency does not exceed 95%.

***Polymorphism information content.

****Heterozygosity observed.

*****Heterozygosity expected.
