# Supplementary material for: Genetic diversity and population structure of Saccharum hybrids
Source: PLoS One. 2023 Aug 15;18(8):e0289504. doi: 10.1371/journal.pone.0289504 (PMC10426985; doi:10.1371/journal.pone.0289504)
Supplement: S3 Table — (DOCX) [file pone.0289504.s004.docx]

**S3 Table**. Diversity indexes for each population.

|  | **Breeding program** | |
| --- | --- | --- |
|  | **Argentinean** | **Brazilian** |
| N^o^ of total amplified bands (TRAP + SSR) | 566 | 571 |
| N^o^ of total polymorphic bands | 527 | 475 |
| % of polymorphism | 93 | 83 |
| Ho | 0.26 | 0.26 |
| He | 0.27 | 0.26 |
| Nei's diversity index | 0.44 | 0.44 |
